# Antimicrobial, Antiparasitic, Anti-Inflammatory, and Cytotoxic Activities of *Lopezia racemosa*

**DOI:** 10.1155/2013/237438

**Published:** 2013-06-12

**Authors:** Carla Cruz Paredes, Paulina Bolívar Balbás, Anaximandro Gómez-Velasco, Zaida Nelly Juárez, Eugenio Sánchez Arreola, Luis Ricardo Hernández, Horacio Bach

**Affiliations:** ^1^Department of Medicine, Division of Infectious Diseases, University of British Columbia, 410-2660 Oak Street, Vancouver, BC, Canada V6H 3Z5; ^2^Departamento de Ciencias Químico-Biológicas, Universidad de las Américas Puebla, Cholula, 72810 Puebla, PUE, Mexico; ^3^Departamento de Ciencias Biológicas, Facultad Biotecnoambiental, Universidad Popular Autónoma del Estado de Puebla, 72410 Puebla, PUE, Mexico

## Abstract

The present study investigates the potential benefits of the Mexican medicinal plant *Lopezia racemosa* (Onagraceae). Extracts and fractions from aerial parts of this plant were assessed to determine their antibacterial, antifungal, antiparasitic, anti-inflammatory and cytotoxic activities *in vitro*. Aerial parts of the plant were extracted with various solvents and fractionated accordingly. Extracts and fractions were tested against a panel of nine bacterial and four fungal species. The antiparasitic activity was tested against *Leishmania donovani*, whereas the anti-inflammatory activity of the compounds was determined by measuring the secretion of interleukin-6 from human-derived macrophages. The same macrophage cell line was used to investigate the cytotoxicity of the compounds. Various extracts and fractions showed antibacterial, antifungal, antiparasitic, and anti-inflammatory activities. The hexanic fraction HF 11-14b was the most interesting fraction with antimicrobial, and anti-inflammatory activities. The benefit of *L. racemosa* as a traditional medicinal plant was confirmed as shown by its antibacterial, antifungal and anti-inflammatory activities. To the best of our knowledge, this is the first study reporting the biological activities of *L. racemosa*, including antiparasitic and anti-inflammatory activities.

## 1. Introduction

*Lopezia racemosa* Cav. (Onagraceae; sin. *L. mexicana*, *L. hirsute* Jacq.) is a plant whose distribution is restricted mainly to México [1]. In Mexican folk medicine, *L. racemosa* has been traditionally used to alleviate stomachache [2], anginas, skin infections, tooth infection, stomach cancer, biliary colic, urine retention [3], and urinary tract infection [3–5]. 

 Chemical profiles from *L. racemosa* have not yet been reported, but diverse polyphenols (e.g., tannins and flavonoids) and sterols have been isolated from the Onagraceae family [6–8]. The main tannin isolated from this family, oenothein B, has both *in vivo* and *in vitro* antitumor activities [9–12]. Among flavonoids, isolated compounds from this family include flavonols, glycoflavones, flavones, and chalcones [13, 14]. Most of the genera studied possess mainly flavonols, which include myricetin, quercetin, kaempferol, and remarkable amounts of various phenolic acids [6, 7, 15]. Furthermore, some species produce significant amount of tocopherols, compounds that have been used as chemotaxonomic markers within the Onagraceae family [16]. 

 Despite its widespread use in México, there is no scientific evidence that supports the abovementioned traditional use. Thus, in the present study, we investigate the antibacterial, antifungal, and antiparasitic activities of *L. racemosa* as well as exploring its cytotoxic and anti-inflammatory effects on human macrophages.

## 2. Materials and Methods

### 2.1. Plant Material

*L. racemosa* was collected at 2476 m above sea level in San Nicolás de los Ranchos, Puebla, México (19°04′03′′ N and 98°29′79′′ W). A voucher specimen numbered 14469 was deposited in the Herbarium of the Benemérita Universidad Autónoma de Puebla, México.

### 2.2. Preparation of Plant Extracts

Extracts from 135 g of air-dried aerial parts from *L. racemosa* were extracted sequentially with the following solvents: *n*-hexane (hexane), chloroform, and methanol. Three rounds of extraction were conducted for each solvent over a period of 48 h. Extracted materials were filtered and the solvents volatilized *in vacuo* at 40°C. The hexane, chloroform, and methanol extracts (HE, CE, and ME) yielded 0.67%, 0.97%, and 24.65% of residue, respectively. The extracts were chromatographed using silica gel (70–240 mesh) and 190 mL of pure or combined solvents. A total of 115 fractions corresponding to all of the three extracts were collected and combined according to their TLC profile, generating five hexanic fractions, four chloroformic fractions, and six methanolic fractions, which were further analyzed for their bioactivity properties. Fractions were dried in a vacuum using a rotary evaporator at 40°C. Stock solutions were prepared by dissolving 20 mg of each extract or fraction in 100 *μ*L DMSO, following sonication (Branson 3210) for 60 min at 30°C until the material was dissolved. pH values varying between 6.5 and 7.3 were measured after stock preparation of extracts and fractions.

### 2.3. Strains and Culture Media

The following Gram-negative strains *Acinetobacter baumannii* (ATCC BAA-747), *Escherichia coli* (ATCC 25922), *Pseudomonas aeruginosa* (ATCC14210), and *Salmonella typhimurium* (ATCC 13311) were assayed. The Gram-positive strains included *Bacillus subtilis* (ATCC 6633), *Staphylococcus aureus* (ATCC 25923), methicillin-resistant *Staphylococcus aureus* (MRSA) (ATCC 700698), and *Streptococcus pyogenes *(ATCC 51878). The acid-alcohol-fast *Mycobacterium smegmatis* mc^2^155 (ATCC 700084) was also included in the panel of bacteria to be tested. The filamentous fungi* Aspergillus fumigatus* (ATCC 1022) and *Trichophyton rubrum* (ATCC 18758), the yeast *Candida albicans* (provided by Vancouver General Hospital, British Columbia, Canada), and *Cryptococcus neoformans* var. *grubii* (kindly provided by Dr. Karen Bartlett, University of British Columbia, BC, Canada) were tested as representatives of pathogenic fungi. The parasite *Leishmania donovani* Sudan strain 2S was assessed for antiparasitic activity of the extracts and fractions and was kindly provided by Dr. Neil Reiner (University of British Columbia,Vancouver, BC, Canada).

 Bacterial strains were cultured in Mueller-Hinton medium (Becton and Dickinson) except for *Mycobacterium smegmatis*, which was cultured in Trypticase Soy broth (Becton and Dickinson) supplemented with 0.05% Tween-80 (Fisher). Bacterial stocks were maintained on the same medium supplemented with 1.5% agar (Becton and Dickinson) at 4°C. All the bacterial strains were cultured at 37°C. For fungal strains, Sabouraud broth (Becton and Dickinson) was used. In the case of filamentous fungi, bioactivities were tested using harvested spores. Spores of *Aspergillus fumigatus* and *Trichophyton rubrum* were carefully harvested and stored as published [17]. Both strains of filamentous fungi were incubated at 28°C. For *Candida albicans* and *Cryptococcus neoformans*, the same protocol used for bacterial strains was followed, but with Sabouraud broth. *Leishmania donovani* promastigotes were cultured as published [17]. 

### 2.4. Antimicrobial and Antiparasitic Assays

A microdilution assay was used to determine the antimicrobial activities in a 96-well plate and according to published protocols [17]. Briefly, bacterial strains were grown overnight by shaking (200 rpm) at 37°C, and the bacterial densities were adjusted to an optical density of 0.05 at 625 nm. Extracts and fractions at concentrations of 10, 40, 100, 200, 400, 500, and 1000 *μ*g/mL were evaluated in a final volume of 200 *μ*L/well. Organisms were incubated for 24 h at 37°C with the exception of fungi, which were cultured at 28°C. The minimal inhibitory concentrations (MICs) were determined when no turbidity in the well was observed. DMSO and untreated inocula were used as negative controls, while amikacin, ampicillin, gentamicin, and rifampicin were used as positive controls. Antifungal activities were assessed in Sabouraud broth (BD) and strains were grown at 28°C for 72 h using the same format of 96-well plates. In the case of *T. rubrum* and *A. fumigatus,* cultures were started from harvested spores as reported in [17]. DMSO and untreated inocula were used as negative controls, while amphotericin B was used as a positive control.

 The evaluation of antiparasitic activity was performed in 24-well flat bottom plates containing 1 × 10^6^ promastigotes/well. Compounds were evaluated at the final concentrations mentioned for the antibacterial activities. Untreated parasites and DMSO were used as negative controls. Motility and number of parasites were annotated at 24, 48, and 72 h after treatment and after staining the sample with 0.4% Trypan blue solution.

### 2.5. Cytotoxic Assay

Human-derived THP-1 monocytic cells (ATCC 202) were cultured in RPMI 1640 (Sigma-Aldrich) supplemented with 5% fetal calf serum (FCS) (Sigma-Aldrich) and 2 mM L-glutamine (STEMCELL Technologies, Vancouver, Canada). THP-1 cells were diluted to a final concentration of 3 × 10^5^ cell/well in a 96-well plate. Plates were placed in a humidified atmosphere at 37°C supplemented with 5% CO_2_ for 24 h. Untreated THP-1 cells and DMSO were used as negative controls, whereas 5% hydrogen peroxide was used as a positive control. Propidium iodide (PI) was used to evaluate membrane damage [18]. The half maximal inhibitory concentration (IC_50_) was calculated by plotting the compound concentrations against the percentage of damaged cells.

### 2.6. Anti-Inflammatory Assay

THP-1 cells at concentrations of 5 × 10^4^ cells/well were dispensed in a 96-well plate and activated with 80 ng/mL phorbol myristate acetate (Sigma-Aldrich). Plates were incubated at 37°C in a humidified atmosphere supplemented with 5% CO_2_ for 24 h. Then, differentiated cells were gently washed with fresh medium (3X) and incubated in presence of the compounds for 6 h. After this time, cells were gently washed again with fresh medium (3X) and an inflammatory response was initiated upon the addition of 100 ng/mL of lipopolysaccharide (LPS) from *E. coli* (Sigma-Aldrich). After 3 h, cells were pelleted by centrifugation at 1000 rpm for 5 min and the supernatants were transferred to a new 96-well plate and kept at −20°C for further analysis. Interleukin-6 (IL-6) (R&D) was used to evaluate the anti-inflammatory activity using a sandwich ELISA. Briefly, 96-well plates were coated with 20 *μ*g human IL-6 overnight at 4°C. The next day, plates were washed with phosphate buffered saline (PBS), supplemented with 0.05% Tween-20 (PBS-T), and blocked with 3% bovine serum albumin (BSA, Sigma-Aldrich) dissolved in PBS overnight at 4°C. BSA was washed away with PBS-T (3X) and 50 *μ*L of each supernatant was added. After 2 h incubation, wells were washed again with PBS-T (3X) and exposed to biotinylated anti-human IL-6 (R&D) according to manufacturer's instructions. After 1 h incubation, wells were washed again with PBS-T (3X) and avidin-HRP (BD Opt EIA) diluted 1 : 250 in 3% BSA was added. After a further 1 h incubation, wells were washed again with PBS-T (3X) and the presence of IL-6 was evaluated by the addition of 50 *μ*L 3,3′,5,5′-tetramethylbenzidine until a blue colour developed. Reactions were stopped by the addition of 25 *μ*L of 1 M sulfuric acid solution. Absorbance was read in an ELISA reader (Bio-Rad) at 450 nm. THP-1 cells exposed to DMSO or LPS were used as negative controls, while dexamethasone was used as a positive control. Values are expressed as percentage of inflammation after normalization to LPS.

### 2.7. Statistical Analysis

A Student's *t*-test was used for statistical analysis. A *P* value <0.05 was considered significant.

## 3. Results

### 3.1. Antimicrobial Activities

 Extracts and fractions obtained from *L. racemosa *were tested against a panel of Gram-negative and -positive bacteria. Four different fractions showed significant activity in inhibiting the growth of bacteria at concentration ranging from 40 to 400 *μ*g/mL (Table 1). Fractions HF 11–14b and MF 28–36 and the extract CE prevented the growth of MRSA at concentrations of 40 *μ*g/mL, whereas the fraction HF 16 inhibited the growth of *S. aureus *at the same concentration (Table 1).

 The antifungal activity of *L. racemosa *was also investigated against four strains of human pathogenic fungi. Only the fractions CF 48–50 and MF 28–36 were highly active against *T. rubrum *at concentration as low as 10 and 40 *μ*g/mL, respectively (Table 1), whereas the fraction MF 17 inhibited the growth of both yeasts *C. albicans *and *C. neoformans *at concentrations of 400 *μ*g/mL.

### 3.2. Antiparasitic Activity

**Figure 1:**
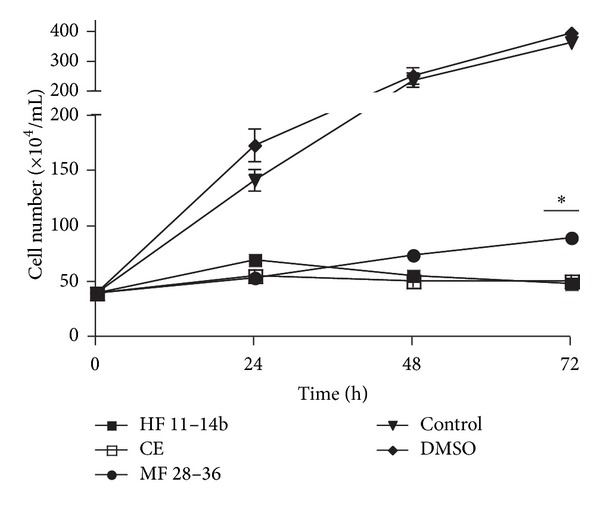
Antiparasitic activity exhibited by fractions of *Lopezia racemosa*. *Leishmania donovani* promastigote growth inhibition was evaluated after incubation of the parasites with extracts and fractions. Untreated promastigotes and DMSO were used as negative controls. Shown is the mean ± SE of three independent experiments. *P value < 0.05. CE, chloroform extract; HF and MF represent hexanic and methanolic fractions, respectively.

 Extracts and fractions were also tested against the parasite *L. donovani*. The fractions HF 11–14b and MF 28–36 and the extract CE significantly reduced the number of *L. donovani *parasites 72 h after infection, compared to the nontreated cells, by approximately 88% (Figure 1).

### 3.3. Cytotoxic and Anti-Inflammatory Activities

**Figure 2:**
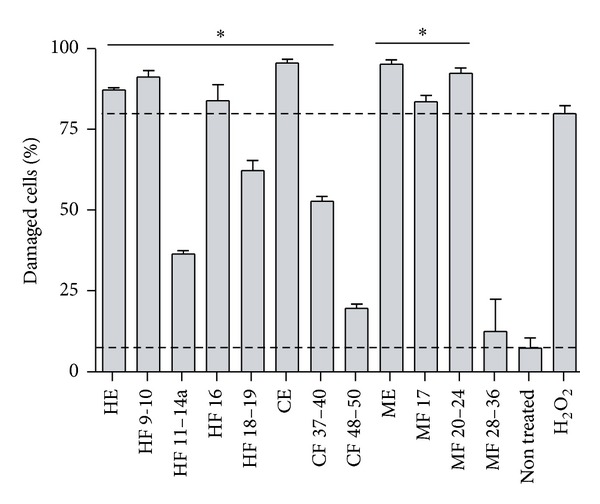
Cytotoxic effects of extracts and fractions of *Lopezia racemosa*. Human macrophages (THP-1 cells) were used to assess the cytotoxic effects of bioactive compounds using propidium iodide staining. Dashed lines represent treatment with 5% H_2_O_2_ (upper line) (positive control), and untreated cells (lower line) (negative control). Shown is the mean ± SE of three independent experiments. *P value < 0.05. HE, CE, and ME are hexane, chloroform, and methanol extracts, respectively. HF, CF, and MF represent hexanic, chloroformic, and methanolic fractions, respectively.

 We assessed the cytotoxic and anti-inflammatory activities of the fractions and extracts that showed either antibacterial, antifungal, or antiparasitic activities. Cytotoxicity was evaluated with human-derived macrophages (THP-1 cells). The majority of extracts showed cytotoxic effects above 50% (Figure 2 and Table 2). Only the fraction MF 28–36 showed no significant cytotoxicity (below 25%) with IC_50_ of approximately 770 *μ*g/mL.

**Figure 3:**
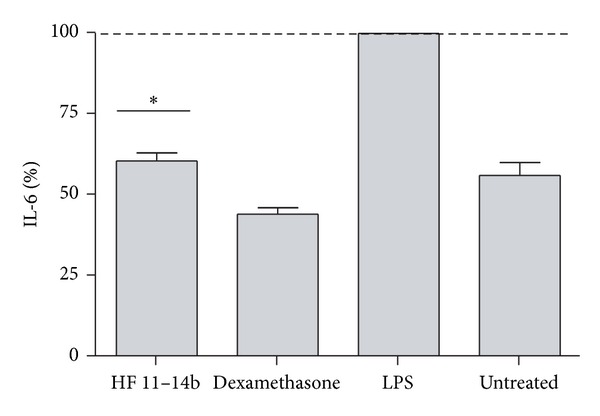
Anti-inflammatory activity of extracts and fractions from *Lopezia racemosa*. An inflammatory process was elicited by exposing macrophages to LPS. Extracts and fractions were exposed overnight to macrophages prior to the addition of LPS. The anti-inflammatory process was evaluated by measuring the secretion of IL-6 into the supernatant. Shown is the mean ± SE of three independent experiments. *P value < 0.05. HF, methanolic fraction.

 The anti-inflammatory activity was assessed by measuring the secretion of IL-6 from macrophages exposed to fractions and extracts. Most of fractions did not possess any anti-inflammatory activity, but fraction HF 11–14b showed significant anti-inflammatory activity by reducing the secretion of IL-6 by approximately 50% when an immunological response was generated by exposure of the cells to LPS (Figure 3).

## 4. Discussion

 In the present study, we examine the biological properties of *L. racemosa*, which has been traditionally used to treat diverse medical conditions such as skin and urinary tract infections as well as stomach cancer. To the best of our knowledge, we provide the first experimental evidence demonstrating its antimicrobial activities against bacteria, fungi, and a parasite.

 Antibacterial activities were found only against the Gram-positive bacteria, MRSA and *S. aureus*. Unfortunately, results from this study cannot be compared to the literature because of the lack of reports about the genus *Lopezia*. However, extracts from different members of the Onagraceae family, such as *Epilobium *and *Ludwigia*, have broad activity against Gram-positive and -negative bacteria [19–22].

 Two fractions of *L. racemosa *showed significant antifungal activity against the dermatophyte *T. rubrum*. In agreement with our study, the extract of *E. angustifolium *has been used clinically to treat tinea capitis, a dermatophytosis of the scalp, which is caused either by *Microsporum *or *Trichophyton *[23]. Several studies have also shown that extracts from *E. angustifolium *were active against a broad range of fungi including several strains of *Candida*, *C. neoformans*, *Saccharomyces cerevisiae*, *Microscoporum canis*, and the saprobic fungus *Rhizopus *sp. [20, 22, 24, 25]. On the other hand, ethanol extract of *Oenothera biennis *was highly active against the phytopathogen *Fusarium semitectum* [26].

 In this study, we report for the first time that two fractions (HF 11–14b and MF 28–36) and one extract (CE) showed significant antiparasitic activity against *L. donovani*.

 Despite their antimicrobial activities, fraction MF 28–36 was not toxic to macrophages. Moreover, a single fraction HF 11–14b showed significant anti-inflammatory activity. Diverse studies have demonstrated that different members of the Onagraceae family have selective anti-inflammatory activity in animal models [7, 8, 15, 27] and cytotoxic activities against several tumor cell lines [11, 12, 28–30].

 It is remarkable that extracts of the members of the Onagraceae family have diverse biological effects. These properties are due to the high content of polyphenols, such as tannins and flavonoids. Phytochemical analyses have shown that oenothein B is the main tannin isolated from *Epilobium *and *Oenothera*, followed by other flavonoids such as myricetin-, quercetin-, and kaempferol-glycosides [6–8]. Due to its high abundance, oenothein B is considered the main compound responsible for the antitumor, antioxidant, antibacterial, antiviral, and phagocytic activities both *in vivo *and *in vitro *[9–11, 31]. Yet, other compounds present in the extracts may exert different biological activities as observed in different studies. In this regard, extracts of *L. racemosa *may have similar active compounds.

## 5. Conclusions

 In summary, we have demonstrated for the first time that extracts of the aerial parts of *L. racemosa *exert antibacterial, antifungal, antiparasitic, anti-inflammatory, and cytotoxic activities. The fraction HF 11–14b deserves further study to elucidate its specific biological properties and identify the compound(s) responsible for its antimicrobial and anti-inflammatory activities.

## Figures and Tables

**Table 1:** Antimicrobial activity of extracts and fractions of *Lopezia racemosa *expressed as MIC (*μ*g/mL).

	Bacteria						Fungi		
	AB	BS	MRSA	MS	SA	SP	CA	CN	TR
HE	NA	NA	NA	NA	400	NA	NA	NA	NA
HF 9-10	NA	NA	400	NA	NA	NA	NA	NA	NA
HF 11–14b	NA	NA	40	NA	NA	NA	NA	NA	NA
HF 16	NA	400	400	NA	40	400	400	NA	NA
HF 18-19	NA	400	NA	NA	NA	400	NA	NA	NA
CE	400	400	40	NA	NA	NA	NA	NA	NA
CF 37–40	NA	NA	NA	400	400	NA	NA	NA	NA
CF 48–50	NA	NA	NA	NA	NA	NA	NA	NA	10
ME	NA	NA	NA	NA	NA	400	NA	NA	NA
MF 17	NA	400	400	NA	400	NA	400	400	NA
MF 20–24	NA	NA	400	NA	400	NA	NA	NA	NA
MF 28–36	NA	NA	40	400	NA	NA	NA	NA	40
Reference	0.1 (Ak)	15 (Ak)	60 (G)	0.7 (R)	1 (G)	(0.7) (Ap)	2 (A)	2 (A)	ND

AB: *Acinetobacter baumannii*; BS: *Bacillus subtilis*; MRSA: methicillin-resistant *Staphylococcus aureus*; MS: *Mycobacterium smegmatis*; SA: *Staphylococcus aureus*; SP: *Streptococcus pyogenes*; CA: *Candida albicans*; CN: *Cryptococcus neoformans*; TR: *Trichophyton rubrum*. NA: no activity detected; HE: hexane extract; CE: chloroform extract; ME: methanol extract; HF: hexanic fraction; CF: chloroformic fraction; MF: methanolic fraction. Experiments were performed in triplicate. A: amphotericin; Ak: amikacin; Ap: ampicillin; G: gentamicin; R: rifampicin; ND: no determined.

**Table 2:** Half-maximal inhibitory concentrations (IC_50_) of *L. racemosa* extracts and fractions expressed in *μ*g/mL.

Extract	IC_50_ (±SD)	Fraction	IC_50_ (±SD)
HE	30.66 ± 6.3	HF 9-10	49.98 ± 5.1
		HF 11–14a	70.02 ± 9.5
		HF 16	34.3 ± 6.9
		HF 18-19	86.93 ± 12.4

CE	28.58 ± 3.6	CF 37–40	86.96 ± 12.0
		CF 48–50	122.3 ± 2.4

ME	29.87 ± 7.7	MF 4–16	25.62 ± 4.9
		MF 17	33.87 ± 3.1
		MF 20–24	40.3 ± 6.3
		MF 28–36	769.54 ± 98.6
